# Gouty arthritis animal models: limitations, challenges, and optimization strategies

**DOI:** 10.3389/fvets.2026.1785462

**Published:** 2026-04-02

**Authors:** Huan Chen, Yu Zhang, Long Chen

**Affiliations:** 1 Department of Spinal Orthopedics, Guangdong Provincial Second Hospital of Traditional Chinese Medicine, Guangzhou, China; 2 The Eighth Clinical Medical College of Guangzhou University of Chinese Medicine, Foshan Hospital of Traditional Chinese Medicine, Guangzhou University of Chinese Medicine, Foshan, China

**Keywords:** animal models, gouty arthritis, hyperuricemia, monosodium urate, tophus

## Abstract

Gouty arthritis (GA) is an inflammatory disease primarily triggered by the deposition of monosodium urate (MSU) crystals in and around the joints. With rising living standards and shifts in dietary patterns, the global incidence of GA is increasing, accompanied by a trend toward earlier onset. Consequently, elucidating its pathogenesis and developing effective treatments are major priorities in clinical research. Animal models of GA serve as an indispensable platform for mechanistic investigation and therapeutic development, especially for novel pharmacological agents. Current animal models of GA are broadly categorized into three types: MSU crystal-induced acute inflammation models, hyperuricemia-combined models, and those designed to simulate chronic gout and tophus formation. While existing approaches successfully replicate the acute inflammatory response characteristic of GA, they remain substantially limited in recapitulating the key features of the chronic disease phase, notably spontaneous tophus formation and persistent synovitis. This article critically examines the defining characteristics and optimal applications of prevailing GA models and proposes targeted strategies for their future optimization.

## 1 Introduction

Gouty arthritis (GA) is one of the most common inflammatory arthritides. With changes in lifestyle and dietary structure, its incidence is gradually increasing, and the age of onset is becoming younger. This disease is primarily caused by purine metabolism disorder and impaired uric acid excretion in the human body, leading to the deposition of monosodium urate (MSU) crystals in and around joint tissues, which manifests clinically as joint swelling and pain. The pathogenesis involves, first, the occurrence of persistent hyperuricemia, followed by the deposition of MSU crystals within the joint, thereby triggering an acute inflammatory cascade centered on the NLRP3 inflammasome (1–3). The pathogenesis and treatment of GA are key focuses of clinical research (4, 5). Animal models of GA provide a crucial platform for studying disease mechanisms and developing therapeutic approaches, particularly for new drug discovery. However, existing models cannot fully replicate the pathological process of human GA, which to some extent limits the development of novel treatments and drugs (6). Based on the pathogenesis and pathological manifestations of GA, an ideal animal model should be capable of simulating the persistent hyperuricemic state observed in humans; the formation and deposition of MSU crystals in and around the joint; an acute, neutrophil-dominated inflammatory response; and the potential for chronic synovitis and tophaceous disease progression (7). Currently, no single model meets all these criteria. This article analyzes the current status of established and emerging models, evaluates their advantages and limitations in mimicking human GA, and suggests potential optimization strategies.

## 2 MSU crystal-induced acute inflammatory models

This type of model directly mimics the terminal inflammatory effector phase of gouty arthritis by exogenously injecting MSU crystals, bypassing the processes of *in vivo* uric acid metabolism and crystal formation. It is currently the most widely used method.

### 2.1 Model construction methods

The model is primarily constructed by injecting a sterile MSU crystal suspension into joint cavities (such as the ankle or knee joint). The classic Coderre model successfully induced acute gouty ankle arthritis by injecting an MSU solution (50 μL, concentration 10–100 mg/mL) into the ankle joint cavity of rats. This model is one of the earliest methods for local joint injection of MSU to induce the disease (8). Subsequently, this technique has been widely applied to construct models in different joints (such as the knee joint) of various animals, including rats, mice, and rabbits (9, 10). For example, Pineda et al. successfully established an acute attack model by injecting a 50 mg/mL MSU crystal suspension into rabbit knee joints. Ultrasonographic evaluation showed that this model induced the characteristic “double contour sign” in 67% of the joints and bright spot-like crystal aggregates in 75% of the joints, effectively replicating the imaging features and intra-articular environment of human acute gout (7).

### 2.2 Pathological and inflammatory features

Animals rapidly developed signs such as joint redness, swelling, heat, pain, and dysfunction within hours after MSU crystal injection (8). In the rabbit knee joint model, a large number of polymorphonuclear cell infiltrations and MSU crystal aggregates were observed in the synovium on day 3 post-injection, with a significant increase in synovial fluid white blood cell count (7). In the mouse air-pouch model, injection of MSU crystals induced neutrophil aggregation (11).

### 2.3 Application advantages and major limitations

#### 2.3.1 Advantages

This model is simple to perform, highly reproducible, and capable of inducing a strong and consistent acute inflammatory response. It serves as an important tool for studying crystal-immune cell interactions, NLRP3 inflammasome activation mechanisms, and for the preclinical screening of acute anti-inflammatory drugs (such as colchicine and its analogs) (12). This type of model can effectively reproduce the inflammatory manifestations of human acute gout attacks.

#### 2.3.2 Limitations

This model has the following major defects: First, it completely fails to simulate the metabolic origin of gout, bypassing the two key initial steps of persistent hyperuricemia and endogenous MSU crystal formation. Therefore, it may not be suitable for evaluating the efficacy of urate-lowering therapies (such as allopurinol and febuxostat) or for studying disease initiation mechanisms (7, 13). Second, although it can induce inflammation dominated by neutrophils under standardized conditions, some experiments have found that the inflammatory cell infiltration pattern may vary, or may be dominated by lymphocytes. This differs from the typical neutrophil-dominated infiltration pattern of human acute gout attacks (14). Furthermore, the acute inflammation induced by the model is usually self-limiting. For example, in the rabbit model, acute inflammation had largely subsided by day 7, and the synovial fluid white blood cell count had also decreased to a non-inflammatory range (4). This makes it difficult to simulate the long-term pathological processes of recurrent attacks, chronic synovitis, and tophus formation seen in human gout (15, 16).

## 3 Hyperuricemia combination models

These models aim to encompass both the metabolic abnormalities and the acute inflammatory manifestations of gout. This is achieved by first inducing systemic hyperuricemia, followed by applying a local injection to induce an inflammatory stimulus.

### 3.1 Model construction methods

The most common method involves intraperitoneal injection of potassium oxonate combined with local joint injection. Potassium oxonate competitively inhibits hepatic uricase, leading to a rapid and transient elevation in serum uric acid levels (17, 18). For example, a novel chronic gout mouse model successfully induced MSU crystal deposition or tophi in up to 37.9% of mice in the paw or ankle joints. This was achieved through daily intraperitoneal injection of potassium oxonate for approximately 4 months, combined with a high-fat diet and local joint injection of acetic acid to promote MSU formation (17).

### 3.2 Pathological and disease features

Model animals exhibit both elevated serum uric acid levels and typical acute joint inflammatory manifestations. The intensity of inflammation may be altered by the background hyperuricemic state. Pathological assessment shows that such models can lead to significantly aggravated arthritis severity, joint cartilage damage, and bone erosion, and allow for the observation of intra-articular MSU crystal deposition or tophus formation.

### 3.3 Application advantages and major limitations

#### 3.3.1 Advantages

This model more closely approximates the common clinical scenario of acute gout attacks occurring in individuals with hyperuricemia. It is suitable for evaluating compounds with both anti-inflammatory and urate-lowering effects (such as benzbromarone) and allows for preliminary exploration of the influence of metabolic state on the inflammatory response. Compared to simple MSU crystal injection models, this type of model can more accurately simulate the pathological features of GA.

#### 3.3.2 Limitations

The induced hyperuricemia is typically pharmacological and transient, failing to reflect the typical chronic persistent hyperuricemia state of human gout. The inducing agent (potassium oxonate) may cause hepatorenal toxicity, interfering with experimental results. The most critical defect is that the core inflammatory trigger of the model still relies on exogenous injection, making it difficult to simulate the core pathological feature of human disease: the spontaneous formation and deposition of endogenous MSU crystals within the joint.

## 4 Chronic gouty arthritis and tophus models

This type of model aims to simulate the pathological process of the chronic stage of GA characterized by tophus formation and destructive arthropathy.

### 4.1 Model construction methods

Repeated MSU injection model: MSU crystals are injected repeatedly into the joint cavities of rodents (e.g., rats, mice) over several days. This artificially creates a persistent or recurrent inflammatory stimulus to induce chronic synovial hyperplasia and tissue damage. Subcutaneous air-pouch model: Sterile air is injected subcutaneously into the back of mice or rats to form an air-pouch structure lined with synovial-like cells. After the pouch wall matures, MSU crystals are injected into the pouch to simulate a localized gouty synovitis environment. Ryckman et al. (11) established a similar model in mice, while Rull et al. (19) and Nalbant et al. (20) established similar models in rats. Avian spontaneous model: Chickens and quails, which naturally lack the enzyme uricase, are used. By feeding them a long-term high-protein or high-purine diet, sustained and stable hyperuricemia is induced. This ultimately leads to spontaneous deposition of MSU crystals (tophi) in joints and internal organs, thereby naturally simulating the chronic disease progression (21, 22).

### 4.2 Pathological and disease features

Repeated injection model: This can lead to persistent synovial hyperplasia and inflammatory cell infiltration in the joint, partially reproducing the histological features of human chronic GA. Air-pouch model: This model can form an inflammatory exudate rich in neutrophils within the pouch cavity, recreating the inflammatory environment of a synovial cavity. Avian model: This model can fully replicate the entire disease course from persistent hyperuricemia to spontaneous deposition of MSU crystals within joints, and ultimately the development of tophi and chronic destructive arthritis. Pathologically, it allows observation not only of typical tophus structure and joint destruction but also key molecular pathological features such as increased xanthine oxidase (XOD) activity, oxidative stress imbalance, and NLRP3 inflammasome activation (21, 22).

### 4.3 Application advantages and major limitations

#### 4.3.1 Advantages

Repeated injection model: The operation is relatively simple, providing a controllable model system for studying chronic joint structural damage caused by repeated crystal deposition. Air-pouch model: It provides a closed, standardized, and easily repeatable synovial-like inflammatory environment (11, 19, 20). Avian model: It is a model capable of simulating the core pathological link of endogenous MSU crystal spontaneous formation and deposition, holding irreplaceable value for studying tophus formation mechanisms. It is particularly suitable for exploring the role of systemic factors like diet and metabolism throughout the disease course and for evaluating the long-term efficacy of urate-lowering therapies in preventing and reversing chronic pathological changes (e.g., tophi and bone erosion) (21, 22).

#### 4.3.2 Limitations

Repeated injection model: It relies on exogenous injection and cannot simulate the complex pathological process of human GA. Air-pouch model: Although it is an excellent system for studying inflammatory responses, its structure is not a real joint. It lacks the complex anatomy (e.g., cartilage, subchondral bone) and physiological biomechanical loading of a joint. Therefore, it cannot simulate or study the structural joint damage crucial to GA, such as cartilage destruction and bone erosion. Avian model: Its immune system, anatomy, physiology, metabolism, and species specificity differ significantly from humans. This limits its application.

## 5 Genetically modified models

This type of model aims to simulate the pathological basis of human hyperuricemia at its genetic root by directly intervening in uric acid metabolic pathways through genetic engineering technology.

### 5.1 Model construction methods

The most representative approach is the construction of urate oxidase (Uox) gene knockout mice. By knocking out the Uox gene, mice lose the ability to decompose uric acid, resulting in persistent hyperuricemia. The early model constructed using homologous recombination technology exhibited severe hyperuricemia and urate nephropathy (23).

Subsequent studies successfully generated a more stable Uox gene knockout mouse model on a C57BL/6J background using the transcription activator-like effector nuclease (TALEN) technique. This model can survive long-term and demonstrates stable hyperuricemia and related metabolic disorders (24). In addition, researchers have constructed a double knockout mouse model targeting both Urat1 (encoding the urate transporter URAT1) and Uox to study specific uric acid metabolism disorders and related renal complications (25).

### 5.2 Pathological and disease features

The core pathological feature of Uox gene knockout mice is severe persistent hyperuricemia, accompanied by extensive renal tubular urate crystal deposition, renal dysfunction, and systemic metabolic abnormalities such as glycol-metabolic disorders, compromised insulin secretion, hypertension, and aberrant lipid metabolism (24). Although this model perfectly simulates the fundamental defect in human uric acid metabolism, spontaneous arthritis is rare, and the formation of joint tophi is inconsistent.

### 5.3 Application advantages and major limitations

#### 5.3.1 Advantages

This model precisely simulates the fundamental genetic defect of hyperuricemia in humans caused by the lack of functional uricase. It is an ideal tool for studying hyperuricemia, related renal complications, and their causal relationship with systemic metabolic disorders (23, 24). The model exhibits high stability and strong expandability. It can serve as a foundational model for evaluating the efficacy of urate-lowering drugs or be combined with other genetic modifications to study specific pathological mechanisms.

#### 5.3.2 Limitations

The model is associated with severe renal disease and lacks typical GA manifestations. Spontaneous joint inflammation and tophus formation are uncommon, making it difficult to directly simulate the joint manifestations of human gout. Species response differences: The physiological response to hyperuricemia differs between mice and humans (e.g., mice are more prone to severe kidney disease), which may limit the clinical translation of some research findings.

## 6 Discussion

Animal models serve as an essential platform for investigating the pathogenesis, therapeutic strategies, and development of novel treatments for GA. Currently, there are four primary approaches for constructing animal models of GA, each with distinct advantages and disadvantages.

The acute inflammatory model involves direct injection of MSU crystals, which rapidly induces typical symptoms such as joint redness, swelling, heat, and pain. This model is suitable for screening anti-inflammatory drugs and studying inflammatory mechanisms. The hyperuricemia model first establishes a hyperuricemic state through pharmacological intervention, followed by crystal injection, making it more clinically relevant. However, the drug-induced metabolic abnormality is often transient and non-physiological. The chronic disease model simulates long-term disease progression either through repeated injections or by utilizing spontaneous pathologies in avian species, allowing observation of tophus formation. Nevertheless, these methods are operationally complex and involve significant species differences. The genetically modified model simulates metabolic defects at the genetic root, providing a novel perspective for studying complications associated with hyperuricemia.

However, all these models share fundamental limitations. The most significant is their inability to fully recapitulate the natural progression of human GA, from metabolic disturbance to spontaneous crystal deposition and ultimately to chronic joint damage. Animal experiments often only mimic isolated aspects of the disease, failing to represent its full spectrum. In particular, the crucial step of spontaneous intra-articular crystal formation remains difficult to achieve in existing mammalian models.

Future research needs to achieve breakthroughs in modeling concepts. The core objective is to develop models capable of simulating key joint pathologies of human GA. This requires the models not only to achieve long-term stable hyperuricemia and spontaneous intra-articular MSU crystal deposition but also to replicate characteristic joint pathological changes. These changes include recurrent acute synovitis, chronic synovial hyperplasia, cartilage erosion, bone destruction, and tophus formation.

To achieve this goal, innovation in modeling methodologies is necessary. One promising strategy involves combining animal strains with stable genetic backgrounds with controlled local joint environmental interventions. Such interventions could include micro-injury, low-grade inflammatory stimulation, or alterations in local physicochemical conditions. The aim is to more specifically induce intra-articular pathological processes, rather than merely systemic metabolic abnormalities.

Concurrently, it is essential to establish a scientific model evaluation system. Beyond monitoring conventional metabolic indicators such as serum uric acid, greater emphasis should be placed on utilizing advanced equipment like high-resolution ultrasound, micro-CT, or animal MRI for monitoring intra-articular crystal deposition, synovial inflammation, and cartilage/bone erosion. Additionally, a multidimensional assessment of joint-specific parameters is required. This includes, for example, joint swelling and functional scoring, analysis of inflammatory cells and cytokines in synovial fluid, and histological scoring of joint tissues.

Different research objectives demand the selection of appropriate models. Mechanistic studies require models with high controllability and reproducibility, efficacy evaluation necessitates models with stability and sensitivity, while translational research requires models that closely approximate human pathological features. Researchers should fully understand the characteristics of each model type and make informed selections based on their specific research needs.

## 7 Conclusion

In conclusion, current animal models of GA still require refinement. Through technological innovation and methodological optimization, there is potential to establish animal models that more accurately simulate the pathological features of the human disease.

## Data Availability

The original contributions presented in the study are included in the article/supplementary material, further inquiries can be directed to the corresponding authors.
